# 

**Published:** 1862-01

**Authors:** 


### INDEX.

*Page.*

A new Caustic for Tooth-ache.......... 43
A few facts in American Dentistry Chronologically arranged, by Dr. J. Richardson.......... 53
A valuable suggestion to those using Rubber-work in their practice, by E. Collins.......... 81
Abscess.......... 100
Acknowledgement.......... 569
Atmospheric Pressure Plates.......... 142
A Proposition.......... 142
Atmospheric Pressure, by W. H. Atkinson.......... 158
Articulating Teeth on Rubber Work.......... 188
Amalgam in England.......... 194
An Agreement made and entered.......... 194
Arsenic, its application and use, by Dr. Wm. A. Pease.......... 197
American Dental Association.......... 235
Anæsthetics in Military Surgery.......... 265
Arnica Hair Wash.......... 320
A little experience with Leeches.......... 339
Arsenic and its compounds, by Geo. Watt.......... 350
Alveolar Abscess.......... 381
A new Dental Depot.......... 385
A new Splint for Fracture of the Jaw.......... 458
A Portable Styptic.......... 459
American Dental Convention.......... 450
A new Drill Stock.......... 482
A new Method of Regular and Constant Coarctation of Parts in Operations for Harelip.......... 511
A Peculiar Case.......... 527
American Journal of Opthalmology.......... 530
A Case Uncontrollable. No. 6.......... 531
Advancement of the Dental Profession, by Dr. A. C. Hawes.......... 537
A Squib.......... 570

Bibliographical.......... 271

Book Notice.......... 290

Becquerrel's Galvanic Apparatus.......... 310

But one kind of Dental Caries.......... 382

Block Teeth.......... 532

Correspondence..........28, 140, 185, 495, 539

Cincinnati Dental Association.......... 144

Cases of Professional Ignorance.......... 172

Constitutional Causes of Caries, by H. H. Black.......... 216

Close of the Volume.......... 568

Concerning the opening of Abscesses.......... 230

Concentration.......... 287

Can't afford it.......... 288

Care of the Teeth, by J. Taft.......... 304

Common Colds.......... 306

Creosote Collodion.......... 319

Careless Writing..............................
Care of the Teeth, by J. Taft.............................. 347
Clinical Notes on Intemperance and its Prevention.............................. 369
Carmi C. Franklin, New York City.............................. 388
Curiosities of Leech Culture.............................. 466
Causes and Cure of Diseases of the feet, with practical suggestions as to their Clothing. 532
Cases in Country Practice.............................. 538

Directory of Hospitals.............................. 571
Died.............................. 572
Dental Associations.............................. 568
ental Periodicals.............................. 28
Dental Meeting at Lafayette.............................. 44
Dental Pereostitis—Treatment, by J. Taft.............................. 70
Dr. Ware on General Therapeutics.............................. 124
Death from Chloroform at the Bellevue Hospital, New York.............................. 175
Directions for Vulcanite Work.............................. 192
Dental Supplies.............................. 195
Dental Depot.............................. 196
Dental Practice,.............................. 196
Dentistry as a Profession.............................. 221
Dental Hospitals.............................. 225
Dentistry as a Profession.............................. 236
Dental Convention.............................. 237
Dental Fees, by J. Taft.............................. 245
Destroying Nerves and filling Cavities, by W. H. Shadran.............................. 298
Dentition and its Derangements, by J. Jacobi, M. D.............................. 312, 363, 498, 552
Deoderizing the Breath by cleansing Teeth.............................. 380
Dr. S. S. Gray, Memphis, Tennessee.............................. 387
Doctrines on Exposed Pulps, by W. H. Atkinson.............................. 445
Dentition and its Derangements.............................. 447, 450
Dialysis.............................. 471
Dental Association.............................. 529

Editorial.............................. 435, 480, 94, 142, 188, 234, 287, 336, 382, 525 5 5
Extracts from the preceedings of the Odontological Society, London.............................. 251
Experiments in Vulcanizing Rubber Compounds.............................. 268
Extracting Teeth, by Dr. W. H. Atkinson.............................. 341

Facial Neuralgia, by W. H. Atkinson.............................. 74, 116
Foil.............................. 289
Facial Paralysis.............................. 467
Fully Prepared.............................. 484
Future of Dentistry, By J. Taft.............................. 492

Gold Foil.............................. 50

Hints on Vulcanite Work, by Geo. E. Snow.............................. 478
History of Dentistry on the Pacific Coast.............................. 495

India Rubber Stoppers for Bottles.............................. 42
Iodine.............................. 90
Improved Water Apparatus for Lathe.............................. 233
India Rubber.............................. 323
Integrity, by W. H. Atkinson.............................. 487

Influence of the times upon the Dental Profession, by J. Taft ........ 485
Is Shaving favorable to health. A Plea for Beards ........ 560
Infants with Teeth at Birth ........ 564

James Robinson, Esq ........ 239

Kerosolene as an Anæsthetic ........ 460

Letter from Philadelphia ........ 140, 185
Local Anæsthesia ........ 445
Logwood as an Antiseptic ........ 461, 481
London Surgeons ........ 477
Local Dental Societies ........ 483
Loverings Etherial Varnish ........ 484

Michigan Dental Association ........ 32
Minutes of the Tenth Annual Meeting of the Ohio Dental College Association ........ 14 4
Metals and Alloys, by Dr. B. Wood ........ 210
McLean's Neuralgic Liniment ........ 325
Metals and Alloys, by Dr. B. Wood ........ 357, 533
Molar Tooth lodged in the Tongue ........ 376
More about Leeches ........ 384
Mr. Wakely, London, England ........ 388
Mechanical Dentistry, by B. W. Franklin of New York ........ 440

Nitrate of Silver ........ 47
New Dental Depot ........ 100
New Applications of Vulcanized Rubber, by Dr. J. Richardson ........ 161
Nursing her wrath to keep it warm ........ 190
Notice of Correspondence ........ 191
New Method of Giving Chloroform ........ 228
Necrosis of Inferior Maxilla, involving the whole bones ........ 260
New and Cheap Method of Ventilation, applicable to any apartment ........ 262
Neuralgia of the Teeth ........ 573
New York Correspondence ........ 543

Our Philadelphia Correspondent ........ 49
On the Intermaxillary Bone aud its relations to Hare-Lip and Cleft Palate ........ 137
Obituary ........ 239, 387
Our duty to our Patients, the people at large and our Profession, by W. H. Atkinson ... 293
On Quackery, by M. H. Mitchell, M. D.. Ithica, O ........ 326
On the Manna of Sinai, and the Manna of Syria ........ 377

Physician's Hand-Book of Practice ........ 569
Plastic Metallic Filling, by Dr. B. Wood ........ 400
Practice of Hardening and Tempering Steel ........ 83
Personal ........ 569
Perchloride of Iron as an escharotic ........ 85
Polishing Plates ........ 87
Peccavi ........ 144
Perodontitis ........ 261
Persulphate of Iron ........ 289
Poisoning by Chloriform ........ 320
Practical Hints, by J. D. White ........ 324
Pivot-Teeth, by W. H. Shadoan ........ 361

Professional Signs and Advertisements.... 385
Phosphorus Necrosis.... 468
Plastic Metallic Fillings.... 481
Promotion.... 484

Questions of a Correspondent.... 338

Removal of a Tumor involving the Parotid and Submaxillary Glands.... 282
Rubber Attachment.... 146
Reading in the Dental Profession.... 179
Rubber Attachment.... 234
Root of a Canine Tooth lodged in the thickness of the lower lip simulating a Cancerous Tumor.... 376
Removal.... 483
Sherwood's New Molar Forceps.... 571
S. Driggs.... 39
Selections....37, 83, 172, 221, 260, 306, 363, 447, 552
Smells and their management, by Dr. Geo. Watt.... 149
Success in the Dental Profession, by C. C. Dills, D. D. S....354, 218
Syphillitic Malformation of Teeth.... 229
Success in the Dental Profession No. 2., by C. C. Dills, D. D. S.... 249
Swaging and finishing Plates, by Ambler Tees.... 307
Solidified Creosote.... 321
Some Uses of Paraffin for Chemical Purposes.... 322
Strange Phenomena in the Human Teeth, by W. H. Shadoan.... 392
Spasms from intestinal irritation and Teething.... 456
Success in the Dental Profession by C. C. Dills.... 487
Some of the effects of Tobacco.... 522
Steam Engine.... 569

To our Subscribers.... 571
The Application of Chemistry to Dentistry, by Charles Wetherill, P. H. D., M. D.... 5
The Warping of Plates, by Dr. G. H. Hydes of Lewiston, Ills.... 27
The Dental College.... 28
The Use of the Battery.... 49
The Method of using Artificial Teeth.... 94
The Vulcanite Patent Cases.... 97
The Wants of the Profession, by J. Taft.... 101
The Deciduous Teeth, by J. Taft.... 107
Therapeutical Application.... 131
The value of Statistical Information to the Dentist.... 135
The uses of Rubber.... 190
Toxicology.... 278
Trochisci Calami.... 281
The influence of Sleep over Disease.... 285
Templars Magazine.... 340
The Extraction of Children's Teeth, by Dr. W. H. Atkinson.... 344
The Ladies Sanitary Association of London.... 375
The New England Farmer.... 387
The proper time to Operate, by W. H. Atkinson.... 389
The best means of advancing Dental Science, by Dr. J. Allen.... 394
The Dental Cosmos.... 482
The Dental Quarterly.... 484
The American Dental Convention.... 567
Theoro-Practical Hints.... 565

Utility of Coffee in Soldier's Diet..... ........................................................ 310

Who are Dentists? by J. Allen, D. D. S........................................ .......... 22
Water, Its History, Characteristics, Hygienic and Therapeutic Uses, by S. W. Francis, A. M., M. D.......................................... ........................................ 37
What the "Louisville Journal" says........................................................ 52
Warranting Dental Operations, by J. Taft...... ............................................ ... 168
Who first suggested Headed Pins for Artificial Teeth ?........................................ 301
Wood's Plastic Metallic Filling ............................................................ 528

Vulcanizing Oven........................................................................ 99
Vulcanite Work.......................................................................... 184

### CONTENTS.

ORIGINAL ESSAYS AND COMMUNICATIONS.

The Application of Chemistry to Dentistry. By CHARLES M. WETHERILL, PH., D., M.D. .. 5

Who are Dentists? By J. ALLEN, D. D. S .......... 22

The Warping of Plates. By DR. J. H. HYDE, of Lewistown, Ill .......... 27

CORRESPONDENCE.

The Dental College .......... 28

The Baltimore College .......... 28

Dental Periodicals .......... 28

S. Driggs .......... 31

PROCEEDINGS OF SOCIETIES..

Michigan Dental Association .......... 32

SELECTIONS.

Water: Its History, Characteristics, Hygienic, and Therapeutic Uses. By S. W. FRANCIS, A. M. M. D., .......... 37
India-Rubber Stoppers for Bottles .......... 42
A New Caustic for Toothache .......... 43

EDITORIAL.

THE

### DENTAL REGISTER,

### OF THE WEST.

Issued Monthly, at $3 a year in advance.

DEVOTED TO THE INTERESTS OF THE DENTAL PROFESSION.

**Edited by J. TAFT and GEO. WATT.**

The Volume commences in January and closes in December.

The Register being issued monthly is always fresh, therefore indispensable to the practitioner who desires to keep posted up with the new inventions and improvements which are daily developed. Neither expense nor effort will be spared to give value and variety to its contents. Articles will be illustrated with well executed engravings, whenever they will add to the interest or assist in explanation.

Its extensive circulation and frequency of issue makes it the BEST DENTAL ADVERTISING MEDIUM in the United States.

☞ CONTRIBUTIONS SOLICITED.

Address Original Communications to Geo. Watt, Xenia, O.

Exchanges to J. Taft, or Dental Register, Cincinnati, O.

Business Communications, to

JNO. T. TOLAND, Publisher and Proprietor,
38 West Fourth Street, Cincinnati, O.

Communications must reach the Editor as early as the 15th, to insure insertion in the next issue.

